# Gas Chromatography–Mass Spectrometry Chemical Profiling of *Commiphora myrrha* Resin Extracts and Evaluation of Larvicidal, Antioxidant, and Cytotoxic Activities

**DOI:** 10.3390/molecules29081778

**Published:** 2024-04-13

**Authors:** Naimah Asid H. Alanazi, Abdullah A. Alamri, Abadi M. Mashlawi, Nujud Almuzaini, Gamal Mohamed, Salama A. Salama

**Affiliations:** 1 Department of Biology, College of Science, University of Hail, Hail 2240, Saudi Arabia; n.alenezy@uoh.edu.sa (N.A.H.A.); n.almuzaini@uoh.edu.sa (N.A.); 2 Physical Sciences Department, College of Science, Jazan University, Jazan 45142, Saudi Arabia; alamri@jazanu.edu.sa; 3 Nanotechnology Research Unit, College of Science, Jazan University, Jazan 45142, Saudi Arabia; 4 Biology Department, College of Science, Jazan University, Jazan 45142, Saudi Arabia; 5 Human Anatomy Department, Faculty of Medicine, Jazan University, Jazan 82817, Saudi Arabia; gmahmed@jazanu.edu.sa

**Keywords:** *Commiphora*, resins, GC/MS, antioxidant, tumor cells, larvicidal activity, *Aedes aegypti*

## Abstract

Plant extracts and essential oils can be alternative environmentally friendly agents to combat pathogenic microbes and malaria vectors. Myrrh is an aromatic oligum resin that is extracted from the stem of *Commiphora* spp. It is used in medicine as an insecticide, cytotoxic, and aromatic. The current study assessed the effect of *Commiphora myrrha* resin extracts on the biological potency of the third larval stage of *Aedes aegypti*, as well as its antioxidant and cytotoxic properties against two types of tumor cells (HepG-2 and Hela cell lines). It also used GC–MS to determine the chemical composition of the *C. myrrha* resin extracts. Fifty components from the extracted plant were tentatively identified using the GC–MS method, with curzerene (33.57%) typically listed as the primary ingredient, but other compounds also make up a significant portion of the mixture, including 1-Methoxy-3,4,5,7-tetramethylnaphthalene (15.50%), β-Elemene (5.80%), 2-Methoxyfuranodiene (5.42%), 2-Isopropyl-4,7-Dimethyl-1-Naphthol (4.71%), and germacrene B (4.35%). The resin extracts obtained from *C. myrrha* exhibited significant efficacy in DPPH antioxidant activity, as evidenced by an IC_50_ value of 26.86 mg/L and a radical scavenging activity percentage of 75.06%. The 50% methanol extract derived from *C. myrrha* resins exhibited heightened potential for anticancer activity. It demonstrated substantial cytotoxicity against HepG-2 and Hela cells, with IC_50_ values of 39.73 and 29.41 µg mL^−1^, respectively. Notably, the extract showed non-cytotoxic activity against WI-38 normal cells, with an IC_50_ value exceeding 100 µg mL^−1^. Moreover, the selectivity index for HepG-2 cancer cells (2.52) was lower compared to Hela cancer cells (3.40). Additionally, MeOH resin extracts were more efficient against the different growth stages of the mosquito *A. aegypti*, with lower LC_50_, LC_90_, and LC_95_ values of 251.83, 923.76, and 1293.35 mg/L, respectively. In comparison to untreated groups (1454 eggs/10 females), the average daily number of eggs deposited (424 eggs/L) decreases at higher doses (1000 mg/L). Finally, we advise continued study into the possible use of *C. myrrha* resins against additional pests that have medical and veterinary value, and novel chemicals from this extract should be isolated and purified for use in medicines.

## 1. Introduction

Many tropical and subtropical plants that attract the attention of scientists and researchers worldwide have been investigated for their potential as eco-friendly natural resources [1,2,3]. Recent studies have concentrated on identifying the biological uses of extracted plants as well as their active chemical components [4,5]. In biological systems, free radicals are continually produced, and when they are in excess, they may severely harm tissues and biomolecules, resulting in a variety of pathological conditions such as aging, cancer, inflammation, Alzheimer’s disease, and cardiovascular illnesses [6,7]. Moreover, interest in antioxidants has grown due to their ability to scavenge free radicals and prevent oxidative damage [8,9].

Mosquito-borne diseases present a significant threat to human health, affecting both people and domesticated animals. Their widespread distribution, increasing vector resistance, and limited vaccine availability highlight the urgent need for new control strategies [10,11,12]. Historically, the high infection rates of dengue fever have had a significant impact on economic growth, leading to lost production and increased costs associated with mosquito control [13]. Chemical pesticides like pyrethroids and organophosphates have been widely used for mosquito control, but their effectiveness is declining due to growing resistance [14,15]. Additionally, these chemicals can harm non-target organisms and the environment [16,17]. 

Many plant-derived chemicals have therapeutic characteristics and have been utilized in traditional medicine for a long time. One of the most important plant families, the Burseraceae, or frankincense, family, is renowned as the primary source of two valuable resins: frankincense (derived from *Boswellia* spp., especially *B. sacra*) and myrrh (derived from *Commiphora* spp., particularly *C. myrrha* and *C. erythraea*). Plants within this family are characterized by their ability to produce aromatic and resinous exudates [18]. Traditional medicinal practices utilize these resins for a diverse array of purposes, including the treatment of bacterial infections, wounds, mouth ulcers, and inflammation. Additionally, these resins are employed in the production of incense [19,20]. The genus *Commiphora*, a member of the Burseraceae family, encompasses approximately 200 species. This genus is distributed from northern Africa to central Asia, with the highest abundance found in northern Saudi Arabia and India [21].

One of the most widely recognized and extensively used herbal products in Saudi Arabia is *Commiphora myrrha* (Botanical name: *C. myrrha* (Nees) Engl./*C. molmol* (Engl.) Engl.; English name: gum myrrh; Arabic name: myrrh or mur Hijaz). It was widely employed in old traditional medicine as a disinfectant and antibacterial agent, as well as to relieve pain [21,22]. The plant may commonly be seen in Jizan, which is a city in the Kingdom of Saudi Arabia on the Red Sea coast. *C. myrrha* is an aromatic oleogum resin that is exuded from the stem of *C. myrrha* [23]. It is a potent antibacterial medication used to treat brucellosis, glandular fever, sinusitis, gingivitis, mouth ulcers, and parasite infections [24,25]. Furthermore, the volatile oils from myrrh and their crude extracts showed a variety of biological actions, including cytotoxic, anesthetic, anti-inflammatory, antiviral, and antibacterial properties [26,27,28,29]. Previous phytochemical investigations have indicated that flavonoids, steroids, and lignans are frequently found in plant stems. Triterpenoids are the main component isolated from resins of the *Commiphora* species [19,30]. Numerous *Commiphora* species’ essential oils have been documented to include monoterpenes, oxygenated sesquiterpenes, and sesquiterpene hydrocarbons [31,32,33]. The development of safe, eco-friendly pesticides and microbial control are crucial for public health. In order to detect bioactive ingredients and allelochemicals from medicinal plant resins, crude extracts from plants like *Commiphora myrrha* are being studied. In the current study, the focus was on evaluating the chemical composition of the resin methanol extract from the Arabian Desert eco-species of *C. myrrha*. This assessment was conducted using gas chromatography–mass-spectrometry, aiming to identify the biochemical components responsible for its biological activity. Through in vitro tests, the study assessed the larvicidal effectiveness of the plant resin against *Aedes aegypti* larvae, along with investigating its antioxidant and cytotoxic properties.

## 2. Results and Discussion

### 2.1. Gas Chromatography–Mass Spectroscopy “GC–MS”

**Figure 1:**
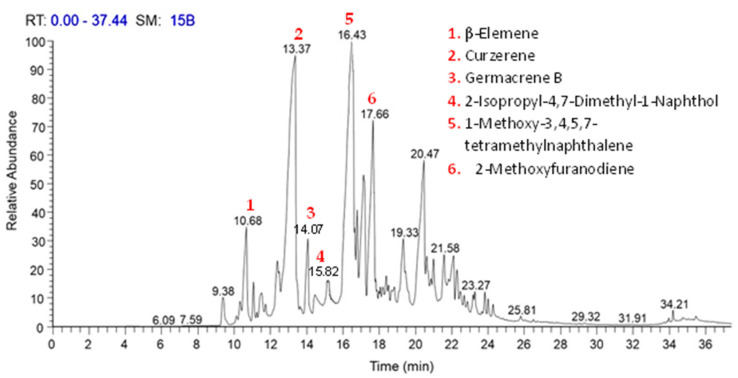
Chromatogram of the methanol extract of *C. myrrha* resins by GC–MS.

The chemical constituents and structural details of the *C. myrrha* resin extracts were determined using gas chromatography–mass-spectrometry. Figure 1 depicts the link between the retention period at which a certain component was detected and the relative quantity of the numerous components identified by the extracted plant. The GC–MS investigation successfully identified 50 constituents, accounting for 100% of the total MeOH extract of *C. myrrha* resin (Table 1). Furthermore, the chemicals that were discovered underwent Electron Ionization (EI) mass spectrometry, and the results may be found in Table S1. Curzerene, identified as the main ingredient, was found to constitute 33.57% of the extract and was eluted at 13.37 min. Other substances were subsequently categorized with higher compositional percentages, including 1-Methoxy-3,4,5,7-tetramethylnaphthalene (15.50%), β-Elemene (5.80%), 2-Methoxyfuranodiene (5.42%), 2-Isopropyl-4,7-Dimethyl-1-Naphthol (4.71%), and germacrene B (4.35%). These compounds represented 69.35% of the total identified compounds (Table 1). The fatty acid derivatives’ components were observed at relatively low abundances, falling between 23.18 and 23.66 min. Meanwhile, the components of diterpenes and triterpenes had retention times ranging from 23.83 to 35.46 min. The most prevalent components of the sesquiterpene were identified within the retention time range of 9.36 to 22.69 min.

It is acknowledged that the diversity or quantity of components differs between the current investigation’s findings and the chemical profile of the previously examined myrrha extract. Nevertheless, some literature reports, such as those by Morteza-Semnani and Saeedi [34], indicate that in the Iranian *C. myrrha* essential oil, curzerene (40.1%), furanoeudesma-1,3-diene (15.0%), and α-elemene (8.4%) are the primary constituents. Similarly, the essential oil of Ethiopian *C. myrrha*, as reported by Marongiu et al. [32], is characterized by furanoeudesma-1,3-diene (38.6%), curzerene (17.5%), lindestrene (14.4%), and α-elemene (4.3%) being the primary constituents.

According to Baser et al. (2003), the primary components of Ethiopian myrrh oil are furanoeudesma-1,3-diene (34.0%), furanodiene (19.7%), and lindestrene (12.0%). Additionally, Mohamed et al. [35] identified major constituents in myrrh oil, including α-elemene (12.86%), 7-isopropyl-1,4-dimethyl-2-azulenol (12.22%), curzerene (11.64%), germacra-1(10)7,11-trien-15-oic acid, 8,12-epoxy-6-hydroxy-ç-lactone (6.20%), δ-elemene (5.57%), δ-neoclovene (5.57%), germacrene B (3.97%), and eremophilene (3.35%). Significantly, the present investigation did not identify the presence of furanoeudesma-1,3-diene, furanoeudesma-1,4-diene, and lindestrene constituents. It is important to consider genetic variables and other environmental impacts during the developmental and growth phases of the wild plant, as these factors may influence the accumulation of active secondary metabolites. Certain chemicals may be created, and their concentrations significantly altered under specific circumstances [36,37].

A significant percentage (93.22%) of the MeOH extract of the *C. myrrha* resin employed in this study was made up of sesquiterpene hydrocarbons. We found there to be 40 separate sesquiterpene components, with curzerene being the bulk (33.57%) of the combined makeup of the isolated components. The current findings validate and expand upon earlier studies, supporting the notion that sesquiterpenoids from the *Commiphora* genus predominantly fall into distinct structural groups. These groups include curzerene, lindestrene, germacrane, eudesmane, guaiane, cadinane, elemane, bisabolane, and oplopane, as previously classified in studies [19,35,38]. The other classes are oxygenated hydrocarbons (3.22%), fatty acid derivatives (1.87%), diterpenes (1.48%), and triterpenes (0.61%) (Table 1). The different climatic conditions in the area, the stage of plant development, and the plant’s adaptive metabolism may be to blame for the variances in the classes of resin extracts [39]. The well-known medicinal benefits and fascinating biological activity of the *Commiphora* plant may be due to the presence of numerous families of sesquiterpenoids in the crude extract and oil of the plant.

### 2.2. Biological Characteristics of the Plant Extracts

#### 2.2.1. Effect of the Plant-Resin Extracts on Larvae

Plant extracts, particularly those rich in phytochemicals, offer a valuable and underutilized resource for mosquito control. Their potential as natural alternatives to synthetic insecticides has been highlighted by numerous studies demonstrating efficacy against mosquito larvae [40,41]. This larvicidal activity is likely linked to the diverse chemical composition of secondary metabolites found in plants. In the present study, we investigated the larvicidal effects of the methanol (MeOH) extract of *C. myrrha* resin against third-instar *Aedes aegypti* larvae. Our results demonstrated a concentration-dependent increase in larval and larval-pupal mortality with increasing exposure time. The highest observed mortality rates were 95% and 98.33% for larvae and larval pupae, respectively (Table 2). However, pupal mortality remained moderate, reaching only 5.0% at 1000 ppm after 24 h (Table 2).

Interestingly, adult emergence was either normal or faster in the control group (untreated; 96.67%) compared to the plant-resin extract treatments, where adult emergence was reduced to 5% and 15% at 1000 ppm and 500 ppm, respectively (Table 2). These findings suggest the potential for the MeOH extract to disrupt mosquito development beyond just larvicidal activity. Our observations align with reports by Baranitharan and Dhanasekaran [42], who found ethyl acetate and chloroform extracts of *Commiphora caudata* resin to be highly effective against larvae of *A. aegypti*, *Anopheles stephensi*, and *Culex quinquefasciatus*. Additionally, the larvicidal activity of oil resins derived from *Commiphora* species has been documented against *Culex pipiens* [43,44] and other mosquito species [45]. Similarly, Mkangara et al. [46] reported the larvicidal activity of petroleum ether, ethyl acetate, and methanol extracts of *C. swynnertonii* against laboratory-reared larvae of *Anopheles gambiae*, *Culex quinquefasciatus*, and *Aedes aegypti.*

The environmental safety of a pesticide is crucial when used against pests and vectors. A pesticide does not have to kill a lot of the target species to be effective [47]. The data given summarize the susceptibility of the third-instar larvae of *A. aegypti* to the tested plant-resin extracts (Table 3). For the 24 h periods in this study, the resin extracts have an LC_50_ of 251.83 mg/L (the median concentration of extract that results in 50% mortality), LC_90_ of 923.76 mg/L (the concentration of extract that results in 90% mortality), and LC_95_ of 1293.35 mg/L (the concentration of extract that results in 95% mortality) (Table 3). The regression line’s slope for the *A. aegypti* population’s in vitro strain of the third larval stage was 2.485 ± 0.212 when resin extracts were employed. Also, the results indicated that the calculated X^2^ (Chi)^2^ was 1.109 in the treatment with the extract. These results are consistent with those obtained by Samwel et al. [45], who used *Commiphora merkeri* oil-resin extracts against *A. aegypti*, *A. gambiae*, and *C. quinquefasciatus* larvae and discovered that the LC_50_ values were 33.79, 31.99, and 17.70 mg/mL, respectively, after 24 h. According to Muturi et al. [44], the LC_50_ values for *Commiphora erythraea* essential oils were 19.05, 22.61, and 29.83 mg/L, whereas the LC_90_ values for *Culex restuans*, *C. pipiens*, and *A. aegypti* were 31.58, 45.04, and 51.58 mg/L, respectively. Furthermore, according to Mkangara et al. [46], the LC_50_ of MeOH extracts from the stem bark of *Commiphora swynnertonii* against *A. aegypti* and *C. quinquefasciatus* demonstrated weak activity after 24 h of exposure, with LC_50_ values of 1235.68 mg/L and 828.13 mg/L, respectively. Similar studies have shown that the resin extracts of *C. molmol* possess pesticide activity against the blowfly *Lucilia sericata* [48], the fowl tick *Argas persicus* [49], and *Culex pipiens* [50]. Therefore, based on the present study, it can be inferred that these previous resin extracts may serve as effective larvicidal agents for controlling populations of *Aedes aegypti*.

*Commiphora* species are widely found across sub-Saharan Africa’s tropical and subtropical regions, including Madagascar, Arabia, Iran, Pakistan, and India. By sealing wounds and assisting in defense against invading insects, illness, herbivory, and infection, the resin serves as the trees’ primary form of protection [51]. According to the current study, the average number of eggs laid is 424 eggs/10 females at 1000 mg/L MeOH resin extract as opposed to untreated groups (1454 eggs/10 females). The maximum hatching (97.00%) was recorded with a 100 mg/L concentration of resin extract, whereas a 1000 mg/L concentration of resin extract resulted in the lowest egg hatching (69.58%), and the fecundity was impacted (29.16%) (Table 4). It was also found that the methanol extract of *C. myrrh* resin influenced the fatality of the egg embryo, reaching 28.54% compared to the untreated groups (1.10%). Sesquiterpenes, polyphenols, aromatic terpenoids, and fatty acids found in the bark extracts of *C. myrrha* resin may have interfered with protein synthesis or the cross-linking of proteins, which might explain the decreased egg hatching seen in the current study. This finding is corroborated by Siddiqui and Alam [52], who reported that fresh extracts from the fruit, leaf, bark, root, and gum inhibited hatching and fecundity in *Meloidogyne incognita*. Similar effects were observed in the cases of the western cherry fruit fly and the peach fruit fly (*Bactrocera zonata*) [53,54].

Finally, our data and those of others support the hypothesis that the effectiveness of *C. molmol* resin extracts against insect pests may be explained by the presence of numerous secondary metabolites, including sesquiterpenes, phenols, aromatic terpenoids, fatty alcohol, curzerene, germacrene, and elemene [19,50,55]. Curzerene is a volatile, aromatic terpenoid found in many herbs and spices, such as *Rhododendron* species, which has been shown to possess anti-inflammatory, antibacterial, acaricidal, and cytotoxicity properties in vitro [56]. As per Muturi et al. [40], both oleo-resin extract and essential oil derived from the genus *Commiphora* demonstrated toxicity against larvae of *A. caspius* and *C. pipiens*. This toxicity was attributed to the presence of bioactive chemical constituents such as curzerene, germacrene, eremophilene, and elemene.

#### 2.2.2. Antioxidant Activity—DPPH Assay

The antioxidant activity of *C. myrrha* resin methanol (MeOH) extract at different concentrations (5–50 mg/L) was assessed by comparing it to ascorbic acid in terms of its ability to scavenge DPPH free radicals. Concentration-dependent scavenging activity was clearly demonstrated. At 50 mg/L, the inhibition percent of MeOH extracts was determined as 75.06% (Table 5). In the findings, the DPPH radical scavenging effects of the standard and plant extracts are shown using half-maximum inhibitory concentration (IC_50_) values. More effective DPPH radical scavenging capability is indicated by a lower IC_50_ value. With an IC_50_ of 26.86 mg/L, the findings, as shown in Table 5, supported the assumption that the resin extract exhibited the strongest antioxidant scavenging activity.

The MeOH extract of *C. myrrha* is rich in sesquiterpenoids, diterpenes, triterpenes, and sterols, which are known for their potential antioxidant properties. These classes of compounds can act as electron donors, potentially contributing to the antioxidant activity by neutralizing free radicals. These compounds serve as electron donors, effectively neutralizing free radicals by converting them into more stable substances and preventing further radical chain reactions. Earlier investigations conducted by Fraternale et al. [57] and Mohamed et al. [35] support this claim, demonstrating that the MeOH and hexane extracts of myrrha resin had the most significant DPPH radical scavenging activity in comparison to its oils. The authors attributed this finding to three specific furano-sesquiterpenoids (myrhone, 3-methoxy-furanogermacradien-6-one, and 2-methoxy-furanogermacren-6-one) found in the MeOH and hexane extracts of *C. myrrha*. These extracts exhibited the most-potent DPPH radical scavenging action. Furthermore, the ability of bioactive compounds, such as phenolics, fatty acids, terpenes, oxygenated hydrocarbons, or carbohydrates, to eliminate or stabilize free radicals is often used to assess their antioxidant activity [3,58]. A further investigation found that the methanol extract of Boswellia sacra resin had a reduced ability to neutralize DPPH radicals compared to the antioxidant activity of the resin essential oil at the same dose [59]. Curcuma and Eugenia uniflora oils have shown significant curzerene antioxidant action against the DPPH radical [60,61].

#### 2.2.3. Cytotoxic Activity 

Currently, cancer is a prominent public health issue with a substantial worldwide influence on both developed and developing nations, and the prevalence of cancer-related factors is increasing [62,63]. An MTT test was used in this study to assess the cytotoxic properties of the produced plant-resin extracts. The materials were examined in vitro against the HepG-2 and Hela cell lines, two types of tumor cells (Table 6). To assess the results of the tested samples in relation to different cancer cells, doxorubicin was used as a guide medicine. The metabolic activity of the cells was evaluated using the strategy of using cellular oxidoreductase enzymes to convert the MTT tetrazolium dye into insoluble formazan, which exhibits a purple hue. The quantity of viable cells is expected to augment during growth, decrease during cytotoxic therapies, and stay constant (or reach a plateau) during cytostatic treatments. In control trials, samples containing only the appropriate amounts of blank solutions were employed, and they had no effect on cell growth. The control sample is advantageous for determining the percentage of cell viability since it yields 100% vitality of healthy cells. Scientific data suggest that phytochemicals possess substantial anticancer capabilities. Around half of the anticancer medications that were authorized between 1940 and 2014 were derived from natural sources [64,65]. The anticancer activities of resin extracts naturally occurring in wild plants against HGC-27, KMBC, PANC-1, CRL-1739, and COLO-205 cell lines have been recently reported [33,66,67].

**Figure 2:**
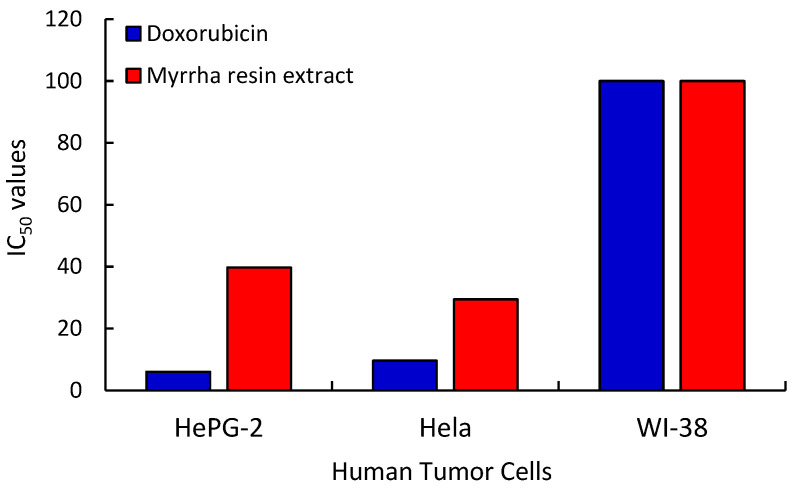
IC_50_ values of the tested plant-resin extracts and doxorubicin as standard against human cancer and normal cells.

Herein, five concentrations of the crude methanolic extracts of plant resin (1.56, 3.125, 6.25, 12.50, 25, 50, and 100 µg/mL) generated by a series of serial dilution were used in the experiments to determine the IC_50_ value (concentration (μg/mL) that causes 50% cell death). On the HepG-2 and Hela cell lines, the % growth inhibition was shown to be steadily increasing with an increasing concentration up to 100 µg mL^−1^. For HepG-2 and Hela human tumor cells and normal cells (WI-38), the extract of *C. myrrha* resin showed inhibitory activities of 94.21%, 92.44%, and 10.11%, respectively. However, among all samples, the lowest concentration (1.56 µg/mL) exhibits the least amount of cytotoxic action (Table 6). The IC_50_ value of this assay for HepG-2 cells pretreated with MeOH extracts of plant resin was 39.73 μg/mL and the R^2^ value was 0.984. In the case of Hela cells, the IC_50_ value was 29.41 μg/mL and the R^2^ value was 0.996. On the other hand, doxorubicin was used as a standard drug to evaluate the effects of the compounds under investigation on the various cancer cells, which produced IC_50_ values for HepG-2, Hela, and WI-38 of 6.03, 9.60, and >100 µg/mL, respectively (Table 6 and Figure 2). These findings are consistent with those presented by Wang et al. [68], who suppressed HGC-27 (human gastric carcinoma cells) cells with an IC_50_ of 27.51 μg/mL to investigate the cytotoxic impact of a sesquiterpenoid from the Kenya ecospecies of *C. myrrha* resin extract. In the current investigation, a significant fraction (93.22%) of the *C. myrrha* resin MeOH extract was made up of sesquiterpene hydrocarbons. In similar work on resin extracts from other plant species, Alipanah and Zareian [69] and Wang et al. [70] discovered that boswellic acid derived from the gum resin of *Boswellia serrata* may effectively suppress the growth of the 4T1 cell line (breast cancer cells) and HCT-116 (colon cancer cells). The IC_50_ values, determined using the MTT test, were found to be 92.3 μg/mL and 15 μg/mL, respectively.

In order to evaluate the toxicity of the plant extract on normal cells and determine its potential for therapeutic use, the selectivity index (SI) was computed. Elevated SI values arise from substantial disparities in the cytotoxicity between cancerous and normal cells, indicating that cancerous cells will experience a more rapid demise compared to normal cells. In this study, the selectivity index values for HepG-2 and Hela cancer cells were 2.52 and 3.40, respectively (Table 6). These results showed that *C. myrrha* resin extract has high cytotoxic selectivity (SI > 1) for human cancer cells, indicating the need for further, in-depth study of the resin extract. For Hela cells, especially high SI values were attained.

The resin obtained from this plant has non-cytotoxic effects on normal cells (WI-38) and shows moderate cytotoxicity against two types of human tumor cells (HepG-2 and Hela), as shown by the IC_50_ values. It is important to note that the toxicity of extracted samples as agents that kill HepG2 and Hela tumor cells is often linked to the structural characteristics (such as size, aggregation, and surface shape) of the components in each extract, as well as the concentration and type of cancer cell line [71]. This result is consistent with the findings of [3] and Salama et al. [72], who investigated the anticancer effects of MeOH extracts derived from *Rumex vesicarius* and *Reichardia tingitana*. The extracts were evaluated in vitro against HepG-2 and PC3 cells using the MTT assay.

## 3. Materials and Methods

### 3.1. Plants Materials

**Figure 3:**
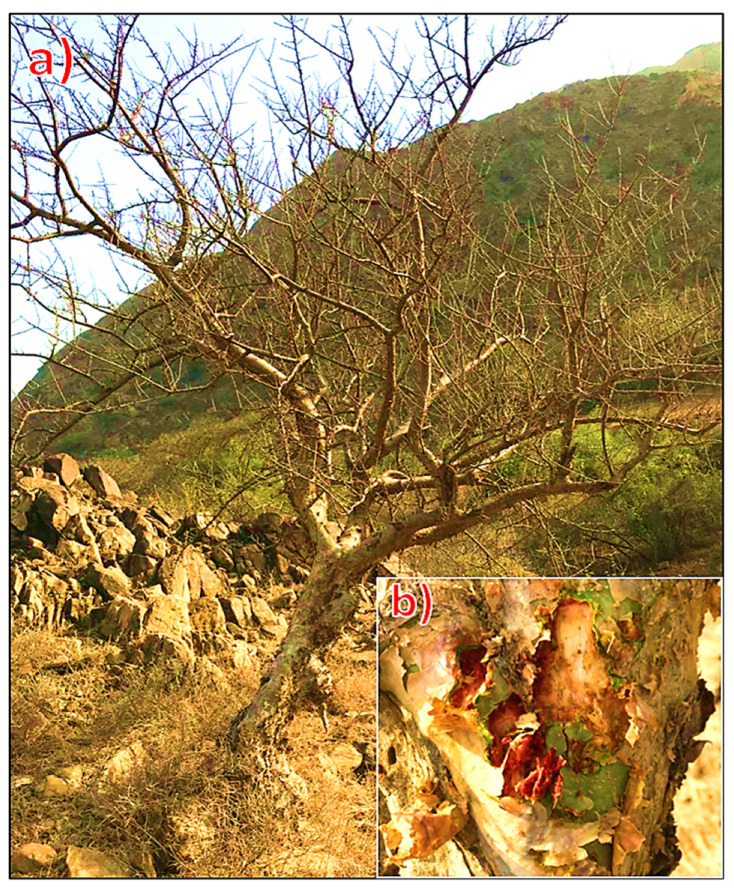
*Commiphora myrrha* (Nees) Engl. (**a**) overview of the growing tree, (**b**) a close-up of tree bark demonstrating resin accumulation from scraping.

The gum resin of the *Commiphora myrrha* (Nees) (Engl. (*C. myrrha)*) tree was gathered in May 2022 from several locations in Wadi Lajab (GPS: 17°35′ N, 42°55′ E), southwest of the Jazan region, Saudi Arabia, by scratching the tree bark (Figure 3). The plant was identified according to Collenette [73]. At the Biology Department, Faculty of Science, Flora and Phytotaxonomic Section, Jazan University, plant resins were identified.

### 3.2. Preparation of Crude Extracts

*Commiphora myrrha* resin (25 g) was ground into a fine powder and macerated separately in glass bottles with 150 mL of methanol. The bottles were shaken for 24 h at room temperature in an orbital shaker (Heidolph^®^ Unimax Orbital Shakers 1010, Schwabach, Germany). Whatman No. 4 filter paper was used to filter the extracts. With new aliquots of the same solvent, residues were extracted twice. To obtain methanolic extract (3.86 g, 15.44%, *w*/*w*), supernatants from each solvent were combined and evaporated under vacuum (Heidolph^TM^ Instruments Hei-VAP) at 40 °C. The obtained crude extracts were utilized for further analysis after being re-dissolved in methanol at a concentration of 1 μg/μL [74].

### 3.3. Gas Chromatography–Mass Spectrometry (GC–MS) Analysis

The analysis of the chemical composition of the extracted resin from *C. myrrha* was conducted using Thermo Scientific capillary gas chromatography (Thermo Scientific, Austin, TX, USA) equipped with a direct capillary column TG-5MS (30 m × 0.25 mm × 0.25 m film thickness) [75]. The temperature of the column oven was initially held constant at 50 °C, then increased at a rate of 5 °C per minute until it reached 250 °C, where it was held for 2 min. Subsequently, it was further increased at a rate of 30 °C per minute to achieve the final temperature of 300 °C, and maintained for 2 min. Temperatures of 260 and 270 °C were maintained for the MS injector and transfer line, respectively. Helium (He) served as the carrier inert gas at a constant flow rate of 1 mL/min.

The remaining sample after solvent evaporation (the concentrated extract) is then injected into the GC using the autosampler AS1300. The EI mass spectrometry data were collected in packed scan mode over the *m*/*z* range of 50–500, with an ionization energy of 70 eV. The ion source temperature was set at 200 °C. The chemical composition of each specific plant material was determined by comparing the mass spectrum data of the various extracted plant components with those in the mass spectrometry databases WILEY 09 and NIST 14. The GC–MS analysis identified five potential components for each observed peak, and the selected structure for the recommended components was determined based on likelihood factors and the fragmentation patterns of the primary structure.

### 3.4. Mosquitocidal Assay

#### 3.4.1. *Aedes aegypti* Colony

*Aedes aegypti* mosquito larvae were obtained from the Medical and Molecular Entomology Section, Biology Department, Faculty of Science, Jazan University, Saudi Arabia, and were utilized in all experiments. The mosquito larvae were cultivated in round enamel plates (24 × 19 × 10 cm) containing 1.5 L of de-chlorinated water. They were fed daily with Tetramin^®^ fish food and powder biscuit. The colony was maintained at 27 ± 2 °C, 75 ± 5% relative humidity, and a 13:11 h (L/D) photoperiod. Adult mosquitoes were provided with a 10% sugar solution. The mosquito larvae were consistently available for testing and were kept under the same laboratory conditions.

#### 3.4.2. Larvicidal Bioassay

The resin extracted from *C. myrrha* for larvicidal activity was dissolved in 0.2 mL of methanol. Various concentration ranges of each extract (100, 200, 300, 400, 500, and 1000 ppm) were prepared to assess its impact on third-instar *A. aegypti* larvae. Plastic cups of 200 mL capacity, containing 100 mL of dechlorinated tap water, were used for the experiment. Twenty mosquito larvae were introduced into plastic containers with different extract concentrations. Typically, three replicates were conducted for each concentration. All plastic cups were then placed in a mosquito colony under controlled conditions, and subsequent mortality was recorded. Control larvae in 100 mL of water were given 0.1 mL of methanol or two drops of Tween 80. Mortality was observed daily until adult emergence, and deceased larvae and pupae were removed from the containers [76]. The percentage of larval death at 24 h post-treatment (PT) was determined using the following equation [77]:

$$\mathrm{Larval}\;\mathrm{mortality}\;\left(\%\right)=\frac{\mathrm{Number}\;\mathrm{of}\;\mathrm{dead}\;\mathrm{larvae}}{\mathrm{Number}\;\mathrm{of}\;\mathrm{treated}\;\mathrm{larvae}}\times 100$$

The count of eggs laid in both treated and untreated cups was conducted using a stereomicroscope [78]. The total number of eggs and the number laid by a single female (fecundity) were recorded. The percentage of egg hatching was calculated using the following formula:

$$\mathrm{Egg}\;\mathrm{hatching}\;\left(\%\right)=\frac{\mathrm{Number}\;\mathrm{of}\;\mathrm{Egg}\;\mathrm{hatching}}{\mathrm{Number}\;\mathrm{of}\;\mathrm{Egg}\;\mathrm{laid}}\times 100$$

Similarly, the following formula was used to get the fecundity percentage:

$$\mathrm{Fecundity}\;\left(\%\right)=\frac{\mathrm{Number}\;\mathrm{of}\;\mathrm{dead}\;\mathrm{larvae}}{\mathrm{Number}\;\mathrm{of}\;\mathrm{treated}\;\mathrm{larvae}}\times 100$$

### 3.5. Antioxidant DPPH Assay

*Commiphora myrrha* resin was diluted from a concentrated solution of the extracted plant in methanol to the desired amounts (5, 10, 20, 30, 40, and 50 mg L^−1^). The DPPH solution (1 mL, 0.135 millimolar) was introduced into each concentration of the prepared sample solution. The measured concentrations in the tested samples were the standard. The specimens were stored under ambient conditions in the absence of light for a duration of 30 min. Subsequently, the optical density at a specific wavelength of 517 nm was determined using a UV/Vis spectrophotometer (Spekol 11 spectrophotometer, analytic Jena AG, Jena, Germany). The antioxidant scavenging activities were quantified by calculating the percentages using the given equation, using a DPPH solution in methanol as the reference.

$$\%\;\mathrm{Inhibition}=\frac{A\;\mathrm{control}\;–\;A\;\mathrm{sample}}{A\;\mathrm{control}}\times 100$$

The approach was implemented with little modifications compared to prior experiments [39,79]. An exponential curve [80] was used to ascertain the inhibitory concentrations (IC50, mg L^−1^) by examining the relationship between the concentration of the sample and the quantity of remaining DPPH radicals.

### 3.6. Cytotoxic Activity Procedure

Hepatocellular carcinoma (HePG-2) and epithelioid cervix carcinoma (Hela) are specific types of human tumor cell lines, and they were acquired from the ATCC, a company specializing in the production of biological goods and vaccines, located in Cairo, Egypt. Doxorubicin is a frequently used chemotherapeutic agent for treating cancer. The chemical reagents used were RPMI-1640 medium, MTT, DMSO (Sigma Co., St. Louis, MO, USA), and fetal bovine serum (FBS; Gibco Life Technologies, Paisley, UK).

The cell growth and cytotoxicity of the extracted *C. myrrha* were evaluated using a standard colorimetric MTT test, following the protocols outlined by Bondock et al. [81]. The conversion of MTT (2-(4,5-dimethylthiazol2-yl)-3,5-diphenyl-2H-tetrazolium bromide) from a yellow color to a purple color was accomplished by the mitochondrial succinate dehydrogenases present in live cells. The cell strains were generated by adding 10% fetal bovine serum to RPMI-1640 medium. Penicillin (100 units/mL) and streptomycin (100 g/mL) antibiotics were given to an incubator containing 5% CO_2_. The cell lines were placed in a 96-well plate with a density of 1.0 × 10^4^ cells per well and kept at a temperature of 37 °C for 48 h in the presence of 5% CO_2_. The cells were first cultivated for 24 h, after which they were subjected to different dosages of the test substances. After a 24 h drug treatment period, a 20 L solution of MTT (5 mg/mL) was added, and the mixture was incubated for an additional 4 h. In order to disperse the formed violet formazan, 100 L of DMSO was then added to each well. A colorimetric study was conducted using a plate reader (EXL 800, New York, NY, USA) to measure the absorbance values at 570 nm. The IC50 values were generated using nonlinear regression (sigmoid type) using the Origin 8.0^®^ software developed by OriginLab Corporation (https://www.originlab.com/; Accessed 27 March 2021). The equation used to calculate the percentage of inhibition in cell growth involves the optical density (OD) values obtained from the control and testing samples, where the OD represents the absorbance readings.

$$\%\;\mathrm{Inhibition}=\frac{\mathrm{OD}\;\mathrm{control}-\mathrm{OD}\;\mathrm{sample}}{\mathrm{OD}\;\mathrm{control}}\times 100$$

In the current study, the selectivity index (SI) was calculated using the following formula:

$$\mathrm{Selectivity}\;\mathrm{index}\;\left(\mathrm{SI}\right)=\frac{{\mathrm{IC}}_{50}\;\mathrm{for}\;\mathrm{normal}\;\mathrm{cell}\;\mathrm{line}\;\mathrm{WI}38}{{\mathrm{IC}}_{50}\;\mathrm{for}\;\mathrm{respective}\;\mathrm{cancerous}\;\mathrm{cell}\;\mathrm{line}}$$

where IC_50_ is the concentration (μg/mL) that causes 50% cell death.

### 3.7. Data Analysis

A one-way analysis of variance (ANOVA) was performed on the biological data, Duncan’s multiple range tests were performed with the help of the computer program Costat var. 6.311 (CoHort Software, Monterey, CA, USA), and Probit analysis was used to determine the potentially fatal values. Each of the studies was repeated three times, and each time there were three replications.

## 4. Conclusions

The global fight against mosquito-borne diseases necessitates the exploration of novel, eco-friendly pest control methods. Natural products, with their vast potential and efficacy, are attracting increasing research interest. This study contributes significantly to this field by investigating the larvicidal activity of a methanol extract from *C. myrrha* resin against *Aedes aegypti* larvae—a previously unreported application. Our investigation employed GC–MS analysis to tentatively identify 50 components within the extract, with sesquiterpene hydrocarbons being the most abundant class (93.22%). Notably, curzerene was the major constituent at 33.57%. While previous studies have documented the cytotoxic, antioxidant, and anti-dengue virus vector properties of *C. myrrha* resins, our focus on the specific larvicidal effect against *A. aegypti* larvae adds a new dimension to the understanding of the resin’s potential applications. This study highlights the novel potential of the MeOH extract of *C. myrrha* resin as a natural mosquito larvicide. Further research is warranted to explore the extract’s efficacy against other mosquito species and potential synergistic interactions with other plant extracts. Additionally, investigating the underlying mechanisms of larvicidal activity and exploring the extract’s safety profile are crucial steps for future development. Overall, this study contributes to the ongoing search for environmentally friendly mosquito-control strategies by pioneering the exploration of *C. myrrha* resin as a natural alternative.

## Figures and Tables

**Table 1:** The characterized chemical components identified from *C. myrrha* resins.

No.	Compound		RT ^a^ (min.)	SI ^b^	Conc.% ^c^	Molecular Weight	Molecular Formula
		Sesquiterpenes hydrocarbons					
1	δ-Elemene		9.36	548	1.47 ± 0.03	204	C_15_H_24_
2	α-Copaene		10.12	819	0.24 ± 0.01	204	C_15_H_24_
3	β-Bourbonene		10.32	674	0.71 ± 0.01	204	C_15_H_24_
4	β-Elemene		10.68	749	5.80 ± 0.08	204	C_15_H_24_
5	Caryophyllene		11.07	565	1.52 ± 0.03	204	C_15_H_24_
6	β-Copaene		11.24	740	0.18 ± 0.01	204	C_15_H_24_
7	γ-Elemene		11.51	792	1.68 ± 0.02	204	C_15_H_24_
8	α-Humulene		11.73	917	0.35 ± 0.01	204	C_15_H_24_
9	Germacrene D		12.39	704	2.35 ± 0.02	204	C_15_H_24_
10	Aromandendrene		12.47	620	0.74 ± 0.01	204	C_15_H_24_
11	γ-Gurjunene		12.64	731	0.11 ± 0.01	204	C_15_H_24_
12	Curzerene		13.37	961	33.57 ± 0.32	216	C_15_H_20_O
13	Cubebol		13.45	529	0.13 ± 0.00	222	C_15_H_26_O
14	α-Elemene		13.50	579	0.22 ± 0.01	204	C_15_H_24_
15	(+)-Ledene		13.64	614	0.14 ± 0.01	204	C_15_H_24_
16	Selina-3,7(11)-diene		13.73	857	0.13 ± 0.01	204	C_15_H_24_
17	Germacrene B		14.07	636	4.35 ± 0.06	204	C_15_H_24_
18	Furanoeudesma-1,4-diene		14.44	581	1.01 ± 0.02	214	C_15_H_18_O
19	Furanoeudesma-1,3-diene		14.98	603	0.08 ± 0.01	214	C_15_H_18_O
20	Lindestrene		15.30	580	0.45 ± 0.01	214	C_15_H_18_O
21	2-Isopropyl-4,7-Dimethyl-1-Naphthol		15.82	535	4.71 ± 0.07	214	C_15_H_18_O
22	1-Methoxy-3,4,5,7-tetramethylnaphthalene		16.43	847	15.50 ± 0.25	214	C_15_H_18_O
23	Furanoelemene		16.65	593	0.49 ± 0.02	216	C_15_H_20_O
24	Eremophilene		16.80	637	2.61 ± 0.12	216	C_15_H_24_
25	Lindera-lactone		17.14	563	0.35 ± 0.01	246	C_16_H_18_O_3_
26	2-Methoxyfuranodiene		17.66	562	5.42 ± 0.08	246	C_16_H_22_O_2_
27	β-Guaiene		17.99	622	0.21 ± 0.01	204	C_15_H_24_
28	Cycloisolongifol-5-ol		18.07	638	0.32 ± 0.02	220	C_15_H_24_O
29	γ-Eudesmol acetate		18.39	536	0.99 ± 0.03	264	C_17_H_28_O_2_
30	Isovalencenol		18.51	684	0.58 ± 0.02	220	C_15_H_24_O
31	12-Methoxy-19-norpodocarpa-8,11,13-triene		18.67	655	0.50 ± 0.02	244	C_17_H_24_O
32	Gazaniolide		19.45	550	0.24 ± 0.01	230	C_15_H_18_O_2_
33	Furosardonin A		20.09	594	0.29 ± 0.01	232	C_15_H_20_O_2_
34	Furosardonin B		20.63	924	1.21 ± 0.03	232	C_15_H_20_O_2_
35	Bohlmann k2631		20.86	713	0.28 ± 0.03	232	C_15_H_20_O_2_
36	6-(3-hydroxyprop-1-en-2-yl)-4,8a-dimethyl-3-oxo-1,2,3,5,6,7,8,8a-octahydronaphthalen-2-yl acetate		21.01	642	1.46 ± 0.02	292	C_17_H_24_O_4_
37	4-a-Methyl-1-methylene-1,2,3,4,4a,9,10,10a-octahydrophenanthrene		21.58	590	1.76 ± 0.03	212	C_16_H_20_
38	8,9-Dehydro-9-vinyl- cycloisolongifolene		21.82	572	0.17 ± 0.01	228	C_17_H_24_
39	Reynosin		22.48	688	0.50 ± 0.02	248	C_15_H_20_O_3_
40	Beta-Doradecin		22.69	556	0.40 ± 0.01	320	C_18_H_24_O_5_
		Oxygenated hydrocarbons					
41	3-Ethyl-2,6-naphthlenediol		17.73	839	1.58 ± 0.02	188	C_12_H_12_O_2_
42	3-Ethyl-6-(Methoxycarbonyl)-2-Naphthol		22.30	703	1.64 ± 0.03	230	C_14_H_14_O_3_
		Fatty acid derivatives					
43	9,12(Z,Z)-Octadecadienoic Acid, methyl ester		23.18	636	0.80 ± 0.02	294	C_19_H_34_O_2_
44	9-Octadecenoic acid, methyl ester		23.28	793	0.99 ± 0.01	296	C_19_H_36_O_2_
45	Octadecenoic acid, methyl ester		23.66	668	0.08 ± 0.01	298	C_19_H_38_O_2_
		Diterpenes					
46	(3E,7E,11E)-1-Isopropyl-4,8,12-trimethylcyclo- tetradeca-3,7,11-trienol		23.83	641	0.89 ± 0.03	290	C_20_H_34_O
47	Isopropyl-1,5,9-trimethyl-15-oxabicyclo [10.2.1]pentadeca-5,9-dien-2-ol		24.01	578	0.59 ± 0.02	306	C_20_H_34_O_2_
		Triterpenes					
48	24-Noroleana-3,12-diene		33.97	597	0.13 ± 0.01	394	C_29_H_46_
49	24-Norursa-3,12-diene		34.21	607	0.34 ± 0.01	394	C_29_H_46_
50	24-Norursa-3,12-dien-11-one		35.46	557	0.14 ± 0.01	408	C_29_H_44_O
	Total identified				100%		
	Sesquiterpenes hydrocarbons				93.22%		
	Oxygenated hydrocarbons				3.22%		
	Fatty acid derivatives				1.87%		
	Diterpenes				1.48%		
	Triterpenes				0.61%		

^a^ retention time, ^b^ similarity index, ^c^ average concentration of three replications ± standard deviation.

**Table 2:** Effects of *C. myrrha* resin MeOH extracts on different growth stages of the mosquito *A. aegypti.*

Conc. (ppm)	Mortality % (Mean ± SE)			Adult Emergence%
	Larval	Pupal	Larval-Pupal	
Control	0.00 ± 0.00 ^F^	3.33 ± 1.67 ^C^	3.33 ± 1.67 ^F^	96.67 ± 1.67 ^A^
100	11.67 ± 1.67 ^E^	3.33 ± 1.91 ^C^	15.00 ± 2.89 ^E^	85.00 ± 5.77 ^B^
200	35.00 ± 5.00 ^D^	8.33 ± 1.47 ^AB^	43.33 ± 4.41 ^D^	56.67 ± 3.33 ^C^
300	56.67 ± 1.67 ^C^	11.66 ± 1.34 ^A^	68.33 ± 1.67 ^C^	31.67 ± 4.41 ^D^
500	73.33 ± 1.67 ^B^	11.67 ± 1.01 ^A^	85.00 ± 2.89 ^B^	15.00 ± 2.89 ^E^
1000	90.00 ± 2.89 ^A^	5.00 ± 0.40 ^BC^	95.00 ± 2.89 ^A^	5.00 ± 2.89 ^F^

Different superscript letters within each treatment (column) express significant variation at a probability level of 0.05 (Duncan’s test).

**Table 3:** Efficacy of methanolic extracts of *C. myrrha* resin on third larval instar of *Aedes aegypti*.

Conc. (mg/L)	Mortality (%)	LC_50_ (Low.–Up.)	LC_90_ (Low.–Up.)	LC_95_ (Low.–Up.)	Slope ± SE	X^2^ (Chi)^2^
Control	0.00 ± 00 ^F^	281.83 (250.40–316.23)	923.76 (760.43–1197.88)	1293.35 (1021.25–1782.63)	2.485 ± 0.212	1.109
100	11.67 ± 1.67 ^E^					
200	35.00 ± 5.00 ^D^					
300	56.67 ± 1.67 ^C^					
500	73.33 ± 1.67 ^B^					
1000	90.00 ± 2.89 ^A^					

Different superscript letters express significant variation at a probability level of 0.05 (Duncan’s test).

**Table 4:** The impact of MeOH extract of *C. myrrha* resin on oviposition, egg hatching (%) and non-hatching eggs and fecundity of *A. Aegypti.*

Conc. (mg/L)	No. of Eggs Laid	Hatching (%)	Fecundity (%)	Non-Hatching (%)	No. of Non-Hatched Eggs	
					Embryo (%)	Non-Embryo (%)
Control	1454.00 ^A^	97.94 ^A^	100.00 ^A^	2.06 ^B^	0.96 ^D^	1.10 ^B^
100	1433.00 ^A^	97.00 ^A^	98.56 ^A^	3.00 ^B^	1.47 ^BC^	1.54 ^B^
200	1386.00 ^AB^	93.22 ^A^	95.32 ^AB^	6.78 ^B^	1.37 ^BC^	5.34 ^AB^
300	1240.00 ^B^	91.69 ^A^	85.28 ^B^	8.31 ^B^	1.29 ^CD^	7.02 ^AB^
500	811.00 ^C^	86.19 ^AB^	55.78 ^C^	13.81 ^AB^	1.73 ^AB^	12.08 ^AB^
1000	424.00 ^D^	69.58 ^B^	29.16 ^D^	30.42 ^A^	1.89 ^A^	28.54 ^A^

Different superscript letters within each treatment (column) express significant variation at a probability level of 0.05 (Duncan’s test).

**Table 5:** DPPH test results showing the percentage of radical scavenging activity and IC_50_ values at different concentrations of methanol extract of *C. myrrha* resin and the standard ascorbic acid.

Treatment	Conc. (mg/L)	Radical Scavenging Activity (%)	IC_50_ (mg/L)
*C. myrrha* resin	5	10.42 ± 0.42 ^F^	26.86
	10	25.79 ± 1.64 ^E^	
	20	44.31 ± 2.37 ^D^	
	30	53.57 ± 2.88 ^C^	
	40	64.34 ± 3.20 ^B^	
	50	75.06 ± 3.72 ^A^	
	LSD_0.05_	1.82 ***	
Ascorbic acid	1	3.11 ± 0.01 ^F^	11.94
	2.5	11.68 ± 0.03 ^E^	
	5	38.87 ± 0.19 ^D^	
	10	48.22 ± 0.51 ^C^	
	15	61.64 ± 1.42 ^B^	
	20	72.91 ± 1.55 ^A^	
	LSD_0.05_	1.36 ***	

Values are average (n = 3) ± standard deviation. Different superscript letters within each treatment (column) express significant variation at a probability level of 0.05 (Duncan’s test). LSD: least significant difference. *** *p* < 0.001.

**Table 6:** The cytotoxic activity and IC50 values of the methanol extract of *C. myrrha* resin were evaluated against tumor and normal cells at various doses, with doxorubicin used as the reference. Hepatocellular carcinoma (HePG-2), Epithelioid cervix carcinoma (Hela) and normal cell (WI-38).

Samples	Conc. (µg/mL)	In Vitro Cytotoxicity		
		HePG-2	Hela	WI-38
Doxorubicin	100	94.21 ± 3.27 ^A^	92.44 ± 3.30 ^A^	10.11 ± 0.41 ^A^
	50	89.52 ± 3.04 ^B^	82.63 ± 2.95 ^B^	8.75 ± 0.31 ^B^
	25	86.36 ± 1.98 ^B^	76.3 ± 2.64 ^C^	6.55 ± 0.23 ^C^
	12.5	69.51 ± 1.72 ^C^	59.21 ± 2.11 ^D^	3.78 ± 0.07 ^D^
	6.25	52.30 ± 1.26 ^D^	42.70 ± 1.53 ^E^	1.87 ± 0.03 ^E^
	3.125	40.13 ± 0.98 ^E^	26.40 ± 0.94 ^F^	0.67 ± 0.02 ^F^
	1.56	26.62 ± 0.37 ^F^	23.91 ± 0.85 ^F^	0.02 ± 0.0 ^F^
	IC_50_	6.03	9.60	>100
*C. myrrha* resin	100	62.83 ± 2.24 ^A^	70.92 ± 2.53 ^A^	1.09 ± 0.04 ^A^
	50	57.91 ± 2.17 ^B^	60.43 ± 2.11 ^B^	0.95 ± 0.03 ^A^
	25	49.18 ± 1.66 ^C^	53.51 ± 1.84 ^C^	0.77 ± 0.03 ^A^
	12.5	39.66 ± 1.52 ^D^	42.64 ± 1.52 ^D^	0.12 ± 0.01 ^B^
	6.25	32.85 ± 1.09 ^E^	32.45 ± 1.03 ^E^	0.00 ^B^
	3.125	25.13 ± 0.87 ^F^	25.70 ± 0.77 ^F^	0.00 ^B^
	1.56	19.81 ± 0.54 ^G^	12.89 ± 0.38 ^G^	0.00 ^B^
	IC_50_	39.73	29.41	>100
	SI	2.52	3.40	-

IC_50_: inhibitory concentration (µg): 1–10 (very strong), 11–20 (strong), 21–50 (moderate), 51–100 (weak), and above 100 (non-cytotoxic). A favorable SI > 1.0 indicates a drug with efficacy against tumor cells greater than the toxicity against normal cells. Different superscript letters within each treatment (column) express significant variation at a probability level of 0.05 (Duncan’s test).

## Data Availability

The data presented in this study are available upon request from the corresponding author.

## Supplementary Materials

The following supporting information can be downloaded at: https://www.mdpi.com/article/10.3390/molecules29081778/s1, Table S1: Electron Ionization (EI) mass spectra of the detected compounds.
