# Supplementary material for: Gas Chromatography–Mass Spectrometry Chemical Profiling of Commiphora myrrha Resin Extracts and Evaluation of Larvicidal, Antioxidant, and Cytotoxic Activities
Source: Molecules. 2024 Apr 13;29(8):1778. doi: 10.3390/molecules29081778 (PMC11051918; doi:10.3390/molecules29081778)
Supplement: Supplementary file 1 [file molecules-29-01778-s001.zip › molecules-2904224-supplementary.pdf]

Gas Chromatography–Mass Spectrometry Chemical Profiling of Commiphora myrrha Resin Extracts and Evaluation of Larvicidal, Antioxidant, and Cytotoxic Activities

Naimah Asid H. Alanazi [1], Abdullah A. Alamri [2,3], Abadi M. Mashlawi [4], Nujud Almuzaini [1], Gamal Mohamed [5] and Salama A. Salama [4,*]

1 Department of Biology, College of Science, University of Hail, Hail 2240, Saudi Arabia; n.alenezy@uoh.edu.sa (N.A.H.A.); n.almuzaini@uoh.edu.sa (N.A.)
2 Physical Sciences Department, College of Science, Jazan University, Jazan 45142, Saudi Arabia; alamri@jazanu.edu.sa
3 Nanotechnology Research Unit, College of Science, Jazan University, Jazan 45142, Saudi Arabia
4 Biology Department, College of Science, Jazan University, Jazan 45142, Saudi Arabia; amashlawi@jazanu.edu.sa
5 Human Anatomy Department, Faculty of Medicine, Jazan University, Jazan 82817, Saudi Arabia; gmahmed@jazanu.edu.sa
* Correspondence: sasalama@jazanu.edu.sa; Tel.: +966-540432176

Supplementary

Table S1: Electron Ionization (EI) mass spectra of the detected compounds.

**No.**	**Compound**	**RT[a] (min.)**	
1	δ-Elemene	9.36	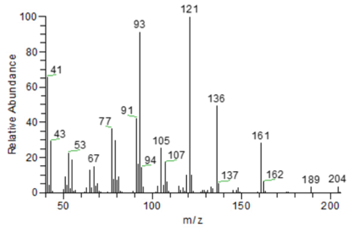
2	α-Copaene	10.12	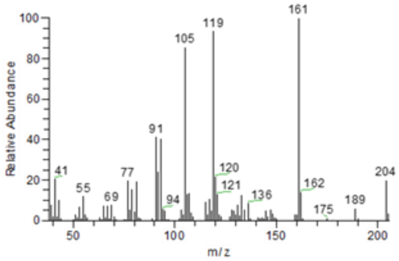
3	β-Bourbonene	10.32	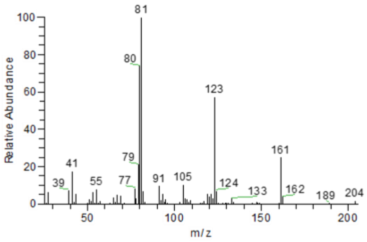

4	β-Elemene	10.68	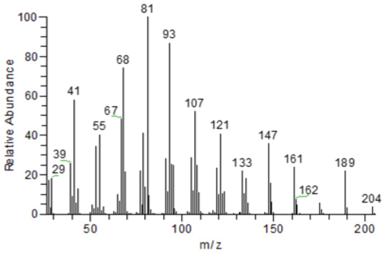
5	Caryophyllene	11.07	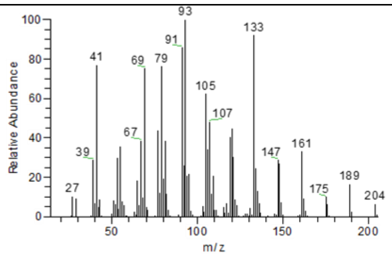
6	β-Copaene	11.24	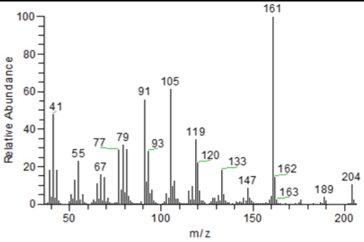
7	γ-Elemene	11.51	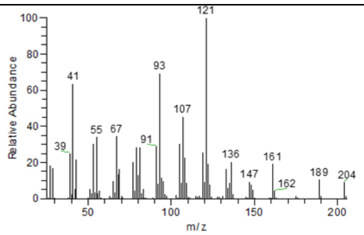
8	α-Humulene	11.73	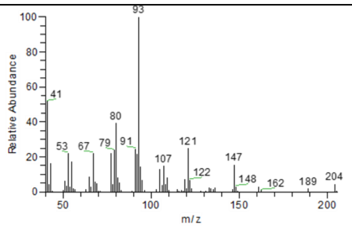
9	Germacrene D	12.39	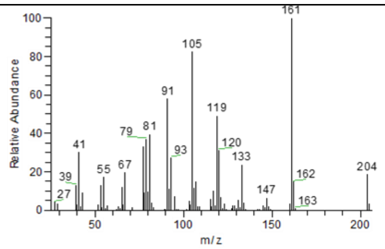

10	Aromandendrene	12.47	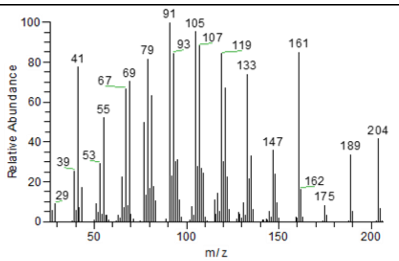
11	γ-Gurjunene	12.64	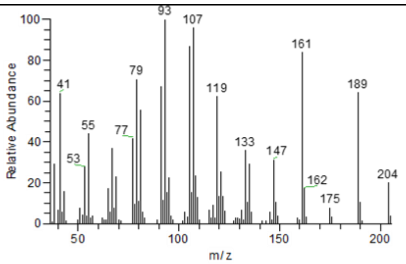
12	Curzerene	13.37	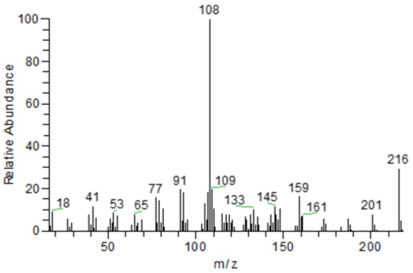
13	Cubebol	13.45	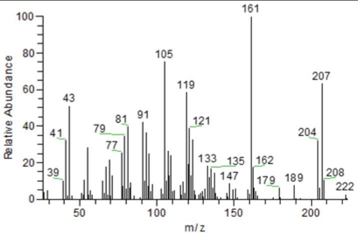
14	α-Elemene	13.50	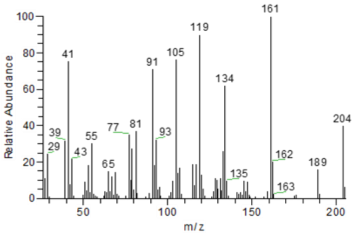
15	(+)-Ledene	13.64	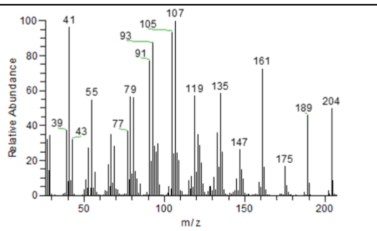

16	Selina-3,7(11)-diene	13.73	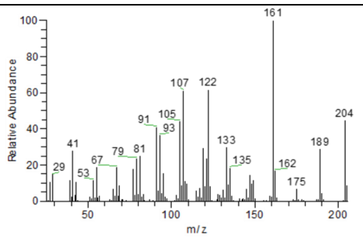
17	Germacrene B	14.07	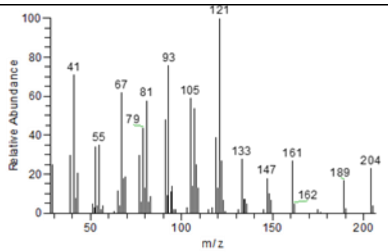
18	Furanoeudesma-1,4-diene	14.44	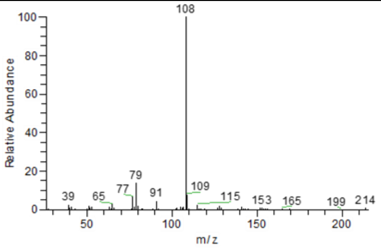
19	Furanoeudesma-1,3-diene	14.98	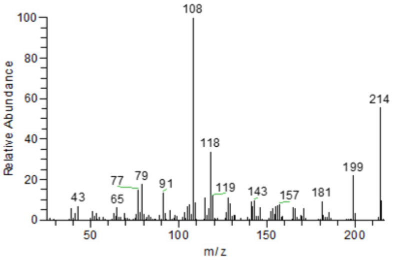
20	Lindestrene	15.30	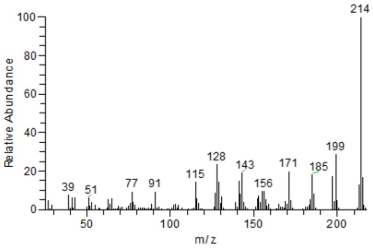
21	2-Isopropyl-4,7-Dimethyl-1-Naphthol	15.82	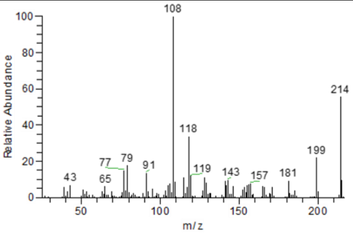

22	1-Methoxy-3,4,5,7-tetramethylnaphthalene	16.43	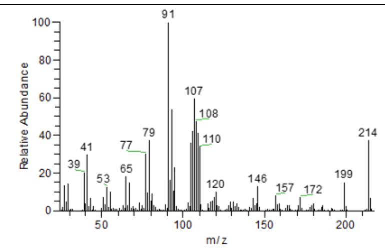
23	Furanoelemene	16.65	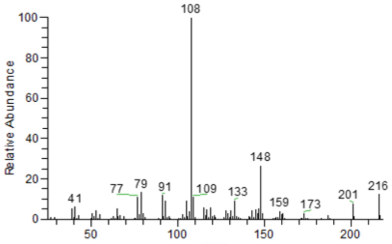
24	Eremophilene	16.80	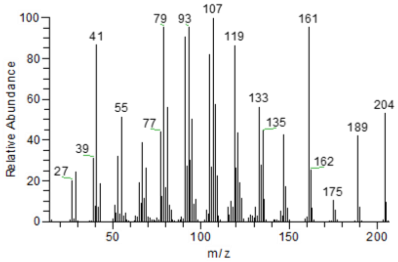
25	Lindera-lactone	17.14	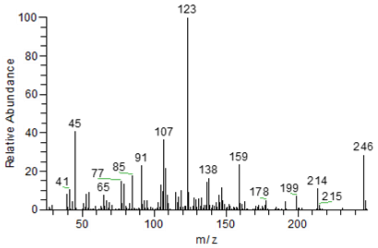
26	2-Methoxyfuranodiene	17.66	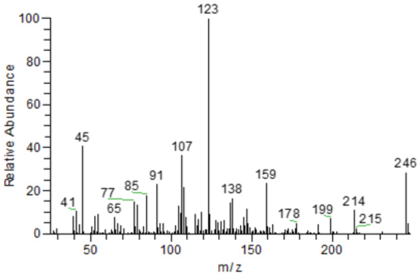
27	β-Guaiene	17.99	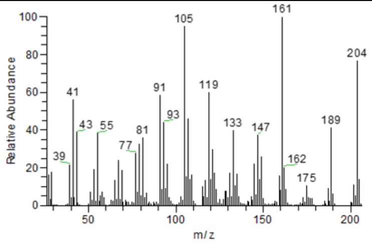

28	Cycloisolongifol-5-ol	18.07	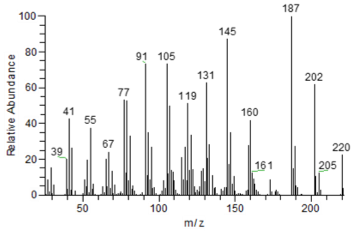
29	γ-Eudesmol acetate	18.39	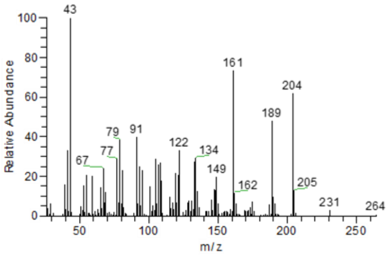
30	Isovalencenol	18.51	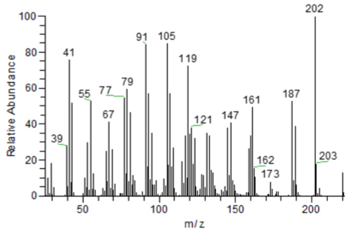
31	12-Methoxy-19-norpodocarpa-8,11,13-triene	18.67	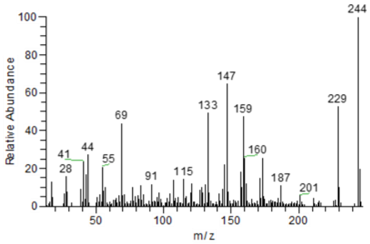
32	Gazaniolide	19.45	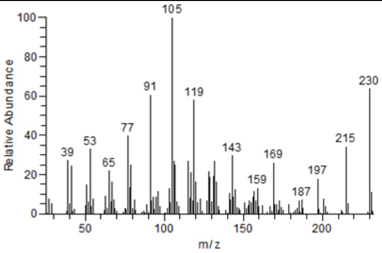
33	Furosardonin A	20.09	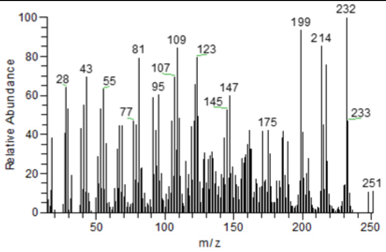

34	Furosardonin B	20.63	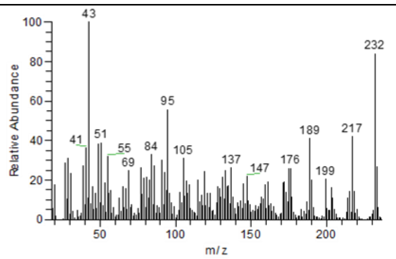
35	Bohlmann k2631	20.86	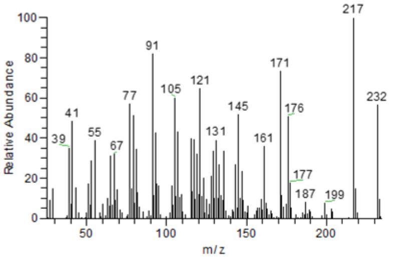
36	6-(3-hydroxyprop-1-en-2-yl)-4,8a-dimethyl-3-oxo-1,2,3,5,6,7,8,8a-octahydronaphthalen-2-yl acetate	21.01	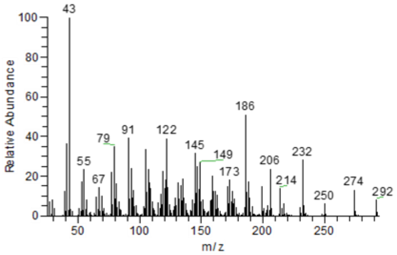
37	4-a-Methyl-1-methylene-1,2,3,4,4a ,9,10,10a-octahydrophenanthrene	21.58	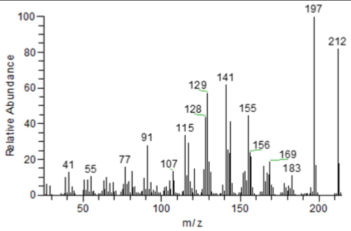
38	8,9-Dehydro-9-vinyl- cycloisolongifolene	21.82	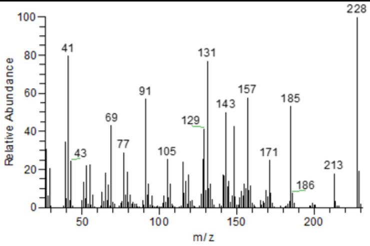
39	Reynosin	22.48	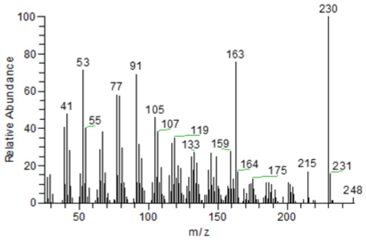

40	beta-Doradecin	22.69	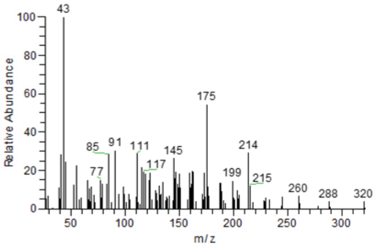
41	3-Ethyl-2,6-naphthlenediol	17.73	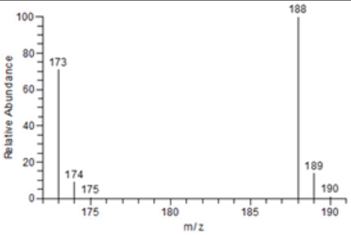
42	3-Ethyl-6-(Methoxycarbonyl)-2-Naphthol	22.30	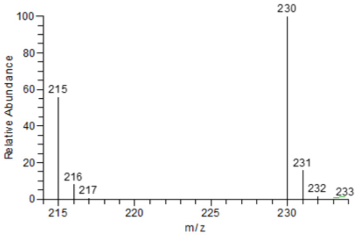
43	9,12(Z,Z)--Octadecadienoic Acid, methyl ester	23.18	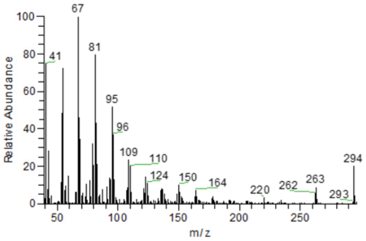
44	9-Octadecenoic acid, methyl ester	23.28	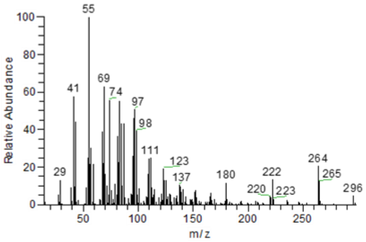
45	Octadecenoic acid, methyl ester	23.66	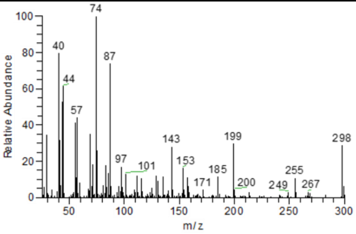
46	(3E,7E,11E)-1-Isopropyl-4,8,12-trimethylcyclo- tetradeca-3,7,11-trienol	23.83	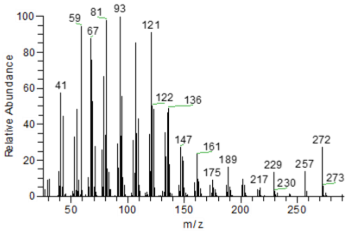

47	Isopropyl-1,5,9-trimethyl-15-oxabicyclo [10.2.1]pentadeca-5,9-dien-2-ol	24.01	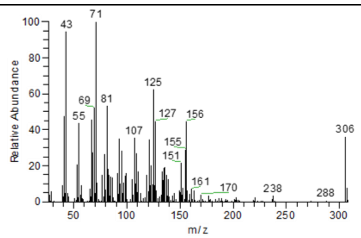
48	24-Noroleana-3,12-diene	33.97	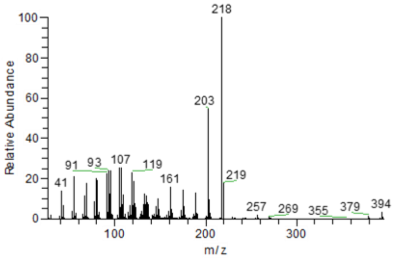
49	24-Norursa-3,12-diene	34.21	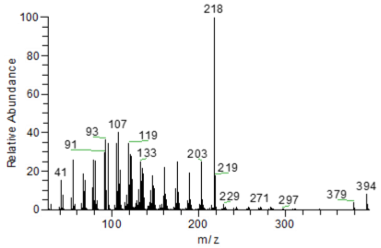
50	24-Norursa-3,12-dien-11-one	35.46	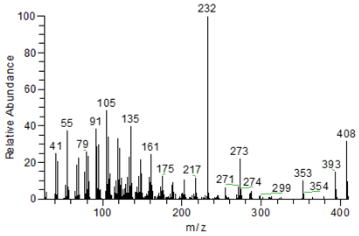
